# Precommissural and postcommissural fornix microstructure in healthy aging and cognition

**DOI:** 10.1177/2398212819899316

**Published:** 2020-01-22

**Authors:** Bethany M. Coad, Emma Craig, Rebecca Louch, John P. Aggleton, Seralynne D. Vann, Claudia Metzler-Baddeley

**Affiliations:** 1 Cardiff University Brain Research Imaging Centre (CUBRIC), Cardiff, UK; 2 School of Psychology, Cardiff University, Cardiff, UK

**Keywords:** Aging, diffusion-weighted magnetic resonance imaging, quantitative magnetization transfer, hippocampus, mammillary bodies, memory, myelin, white matter

## Abstract

The fornix is a key tract of the hippocampal formation, whose status is presumed to contribute to age-related cognitive decline. The precommissural and postcommissural fornix subdivisions form respective basal forebrain/frontal and diencephalic networks that may differentially affect aging and cognition. We employed multi-parametric magnetic resonance imaging (MRI) including neurite orientation density and dispersion imaging, quantitative magnetization transfer (qMT), and T_1_-relaxometry MRI to investigate the microstructural properties of these fornix subdivisions and their relationship with aging and cognition in 149 asymptomatic participants (38–71 years). Aging was associated with increased free water signal and reductions in myelin-sensitive R_1_ and qMT indices but no apparent axon density differences in both precommissural and postcommissural fibers. Precommissural relative to postcommissural fibers showed a distinct microstructural pattern characterised by larger free water signal and axon orientation dispersion, with lower apparent myelin and axon density. Furthermore, differences in postcommissural microstructure were related to performance differences in object-location paired-associate learning. These results provide novel in vivo neuroimaging evidence for distinct microstructural properties of precommissural and postcommissural fibers that are consistent with their anatomy as found in axonal tracer studies, as well as for a contribution of postcommissural fibers to the learning of spatial configurations.

## Introduction

Understanding the underlying causes of age-related memory decline remains a priority. One approach is to relate individual variations in cognition with variations in the microstructure of either grey or white matter. This study adopted the latter approach, focussing on hippocampal formation connections, reflecting the consensus view that the hippocampal formation and its pathways comprise a vital hub for age-sensitive forms of memory (Erickson and Barnes, 2003; Mitchell et al., 2000; Sexton et al., 2010). We, therefore, examined microstructural properties of the fornix, the principal pathway by which the hippocampus communicates directly with sites beyond the temporal lobe (Bubb et al., 2017; Poletti and Creswell, 1977).

Diffusion tensor imaging (DTI) provides a means to explore fornix status and aging. This technique, which uses the diffusion of water to derive indirect measures of white matter microstructure (Jones, 2010; Jones and Leemans, 2011), has repeatedly revealed fornix alterations with healthy aging (e.g. Douet and Chang, 2015; Jang et al., 2011; Ly et al., 2016; Metzler-Baddeley et al., 2011, 2012, 2019a; Sasson et al., 2010). Notably, aspects of memory including recall (Gazes et al., 2019; Metzler-Baddeley et al., 2011, 2012; Zamroziewicz et al., 2017) and working memory (Zahr et al., 2009) have been variously related to DTI-based fornix measures, including fractional anisotropy (FA) and mean diffusivity (MD).

Previous studies have typically treated the fornix as a unitary tract. In fact, it is a complex, bidirectional pathway with two main subdivisions, the precommissural fornix and the postcommissural fornix. While the precommissural fornix innervates orbital and medial prefrontal cortices and carries projections both to and from the basal forebrain, the postcommissural fornix innervates the anterior thalamus and hypothalamus, including the mammillary bodies (Aggleton et al., 2015; Dillingham et al., 2015; Mathiasen et al., 2019; Poletti and Creswell, 1977). Given how the prefrontal cortex is particularly sensitive to aging (Lemaitre et al., 2012; Raz et al., 1997) and the basal forebrain shows age-related neuronal loss (McGeer et al., 1984), it would be expected that white matter pathways connecting these structures, that is, precommissural fornix, would be similarly affected by age. We would expect such age-related differences in the precommissural fornix to be accompanied by impairments in frontal-mediated executive functions. The predictions are not so clear for postcommissural fornix. There is no evidence of age-related neuronal loss in the mammillary bodies but some suggestion of white matter–related atrophy that could reflect a loss of fornix inputs (Wilkinson and Davies, 1978; but see Begega et al., 1999). Two studies have examined the effects of aging on precommissural and postcommissural fornix. The first (20 females, age 23–66 years) found no age-related changes for FA, axial diffusivity (AD), or radial diffusivity (RD) in either fornix subdivision (Chen et al., 2015). A second study (44 participants, age 53–93 years) did find age-related correlations for MD, AD, and RD in the postcommissural fornix (Christiansen et al., 2016). As such, there is no clear consensus as to how these tracts are affected by aging and how any such changes may relate to age-related differences in cognition.

This study compared the precommissural and postcommissural fornix by moving beyond previously applied DTI analyses (Christiansen et al., 2016) to examine white matter microstructural components in more detail in a larger cohort of 149 healthy individuals (age 38–71 years). The additional analyses involved indices of apparent axon microstructure from multi-component diffusion-based neurite orientation dispersion and density imaging (NODDI; Zhang et al., 2012) alongside indices from quantitative magnetization transfer (qMT; Sled, 2018) and T_1_-relaxometry (see section ‘Methods’) that are more sensitive to myelin in white matter than diffusion-based metrics. Furthermore, a comprehensive battery of cognitive tasks helped to determine whether memory functions are more closely aligned with hippocampal–diencephalic connections and, hence, with postcommissural fornix, while executive control is more closely aligned with prefrontal cortex and, hence, precommissural fornix (Christiansen et al., 2016; Williams et al., 2019).

## Methods

This study was approved by the Cardiff University School of Psychology Research Ethics Committee (EC.14.09.09.3843R2). In accordance with the World Medical Association Declaration of Helsinki, all participants provided written informed consent.

### Participants

Participants were community-dwelling individuals recruited from the Cardiff University School of Psychology’s community panel, employee notice board, Internet, and poster advertisements, as part of the Cardiff Ageing and Risk of Dementia Study (CARDS; Metzler-Baddeley et al., 2019a, 2019b). Participants were required to have a good command of the English language and no history of neurological disease (e.g. Parkinson’s disease, Huntingdon’s disease, multiple sclerosis), psychiatric disease (e.g. major depressive disorder, bipolar disorder, schizophrenia), or substance dependency. Participants were also excluded if they had suffered a moderate to severe head injury with loss of consciousness, had high risk of cardio-embolic incidents (e.g. severe heart failure, cardiac aneurysm, aortic stenosis), significant large-vessel disease, or magnetic resonance imaging (MRI) contraindications (e.g. pacemaker, stents).

Demographic and health information about general and lifestyle risk factors for dementia were collected from 211 volunteers, of which 166 went on to undergo cognitive testing and MRI scanning at the Cardiff University Brain Research Centre (CUBRIC). The mean age of participants was 55.8 years (SD = 8.1) with a range of 38–71 years. The participants, of whom 94 were female, had a mean of 16.6 (SD = 3.3) years of full-time education.

### Cognitive assessment

The National Adult Reading Test (NART; Nelson, 1991) was administered to acquire a basic measure of verbal intelligence while the Mini Mental State Examination (MMSE; Folstein et al., 1975) was used to screen for cognitive impairment. Participants were asymptomatic (MMSE: M = 29.1, SD = 1) and scored at a slightly above mean levels of intelligence according to the NART (M = 116.7, SD = 6.6).

Immediate and delayed (30 min) verbal and visual recall was assessed with the Rey Auditory Verbal Learning Test (RAVLT; Rey, 1941) and the complex Rey figure (Schmidt, 1996). Short-term topographical memory was measured with the Four Mountains Test (Chan et al., 2016). Spatial navigation was assessed with a virtual Morris Water Maze Task (vMWMT; Hamilton et al., 2009) that required participants to find a hidden platform in a water pool. This task comprised six blocks of four trials each. The first block was a practise block to familiarise participants with the task. Blocks 2–5 were the experimental blocks where participants had to navigate to a hidden platform. Block 6 was a motor control condition, where participants navigated to a visible platform. Outcome measures for the vMWMT were mean total latencies, first move latencies, and total path lengths for each block. In addition, participants completed a battery of computerised assessments from the Cambridge Brain Sciences laboratory (www.cambridgebrainsciences.com; Hampshire et al., 2012; Owen et al., 2010), designed to be sensitive to working memory and executive function. This battery involved an adapted version of the Stroop test, assessments of digit and spatial span, intra-dimensional and extra-dimensional (IDED) attention set shift, deductive grammatical reasoning, as well as tests of spatial imagery and planning (Hampshire Trees; self-ordered spatial tasks) and the learning of the location of particular objects on a screen (paired-associate object-in-location learning (PAL)). Outcome measures for the Cambridge Brain Sciences tasks were response latencies and mean and maximum number of correct responses.

### Multi-parametric MRI scanning protocol

MRI data were acquired at CUBRIC on a 3T MAGNETOM Prisma clinical scanner (Siemens Healthcare, Erlangen, Germany) utilising a 32-channel receive-only head coil. The MRI acquisition and preprocessing protocols have been published (Metzler-Baddeley et al., 2019a, 2019b). Whole-brain High Angular Resolution Diffusion Imaging (HARDI; Tuch et al., 2002) data were collected using a spin-echo echo-planar dual-shell sequence with diffusion encoded along 90 isotropically distributed directions (30 directions at b-value = 1200 s/mm^2^, 60 directions at b-value = 2300 s/mm^2^). An additional six non-diffusion-weighted scans were acquired with dynamic field correction with the following parameters: repetition time (TR) = 9400 ms, echo time (TE) = 67 ms, 80 slices, 2 mm slice thickness, field of view (FOV) = 256 mm × 256 mm × 160 mm, GeneRalized Autocalibrating Partial Parallel Acquisition (GRAPPA) acceleration factor = 2, acquisition time: ~15 min.

High-resolution T_1_-weighted anatomical images were acquired with a three-dimensional (3D) magnetization-prepared rapid gradient-echo (MP-RAGE) sequence comprising 176 slices with the following parameters: TR = 2300 ms, TE = 3.06 ms, TI = 850 ms, flip angle θ = 9°, 1 mm slice thickness, FOV = 256 mm, acquisition time: ~6 min.

To acquire metrics in relation to myelin, an optimised 3D MT-weighted gradient-recalled echo sequence (GRE; Cercignani and Alexander, 2006) with the following parameters was used: TR = 32 ms, transthoracic echocardiography (TTE) = 2.46 ms, Gaussian MT pulse duration t = 12.8 ms, FA = 5°, FOV = 24 cm, 2.5 mm × 2.5 mm × 2.5 mm resolution. The off-resonance irradiation frequencies (Θ) and their corresponding saturation pulse nominal flip angles (ΔSAT) for the 11 MT-weighted images were optimised using the Cramer–Rao lower bound optimization. They were as follows: Θ = [1000, 1000, 2750, 2768, 2790, 2890, 1000, 1000, 12,060, 47,180, 56,360] Hz and their corresponding ΔSAT values = [332°, 333°, 628°, 628°, 628°, 628°, 628°, 628°, 628°, 628°, 332°]. The longitudinal relaxation time, T_1_, of the system was estimated by acquiring three 3D (GRE) volumes with three different flip angles (θ = 3°, 7°, 15°) using the same acquisition parameters as employed in the MT-weighted sequence (TR = 32 ms, TE = 2.46 ms, FOV = 24 cm, 2.5 mm × 2.5 mm × 2.5 mm resolution). Data for computing the static magnetic field (B_0_) were collected using two 3D GRE volumes with different echo-times (TE = 4.92 and 7.38 ms, respectively; TR = 330 ms; FOV = 240 mm; slice thickness: 2.5 mm; Jezzard and Balaban, 1995).

### MRI data processing and white matter microstructural indices

The two-shell diffusion-weighted HARDI data were split into b = 1200 and 2400 s/mm^2^ data and were separately corrected for artefacts due to diffusion-weighted induced gradients and head motion in ExploreDTI (Version 4.8.3 Leemans et al., 2009) using appropriate reorientation of the encoding vectors (Leemans and Jones, 2009). To correct for echo-planar imaging (EPI)-induced geometrical distortions, the diffusion-weighted image volumes were warped to down-sampled T_1_-weighted images (1.5 mm × 1.5 mm × 1.5 mm; Irfanoglu et al., 2012). After preprocessing, the NODDI model (Zhang et al., 2012) was fitted to the dual-shell HARDI data with the fast, linear model fitting algorithms of the Accelerated Microstructure Imaging via Convex Optimization (AMICO) framework (Daducci et al., 2015) to gain the following diffusion signal components as estimates of axonal microstructure: hindered diffusion (FA and MD), restricted diffusion (intracellular signal fraction (ICSF)), free water (isotropic signal fraction (ISOSF)), and axon orientation dispersion (orientation dispersion index (ODI)).

The qMT-based metrics were the macromolecular proton fraction (MPF) and the forward exchange rate *k_f_* (Sled, 2018) as well as the longitudinal relaxation rate R_1_ (1/T_1_) from T_1_-relaxometry (Callaghan et al., 2015; Rooney et al., 2007). MPF and R_1_ are sensitive to the myelin content in white matter (Callaghan et al., 2015; Ceckler et al., 1992; Koenig, 1991; Levesque et al., 2010; Rooney et al., 2007; Schmierer et al., 2007; Serres et al., 2009) while *k_f_*, an index of the rate of the magnetization transfer process (Sled, 2018), has been proposed to reflect metabolic efficiency of mitochondrial function (Giulietti et al., 2012) and has been shown to be sensitive to neuroinflammation (Harrison et al., 2015).

MPF, *k_f_*, and R_1_-maps were obtained by first co-registering each participant’s MT-weighted GRE volumes to the MT-volume with the most contrast using a rigid body (6 degrees of freedom) registration using Elastix (Klein et al., 2020) to correct for inter-scan motion. To obtain these maps, the 11 MT-Weighted GRE images and T_1_-maps were then modelled by Ramani’s two pool pulsed MT approximation (Ramani et al., 2002). To remove voxels where apparent data appeared solely due to noise, FMRIB’s fslmaths programme was used to threshold MPF maps to an upper intensity limit of 0.3 and *k_f_* maps to an upper limit of 3.

Finally, all whole-brain microstructural metric maps were spatially aligned to the down-sampled reference image of the T_1_-weighted anatomical volume in within-subject space using linear affine registration with 12 degrees of freedom in FMRIB’s Linear Image Registration Tool (FLIRT).

### Tractography

The RESDORE algorithm (Parker, 2014) identified outliers within the HARDI data. Following this, whole brain tractography was completed using in-house software (Parker, 2014) for each subject on the 60 direction b = 2400 s/mm^2^ HARDI data using the damped Richardson–Lucy (dRL) algorithm (Dell’acqua et al., 2010). For tract reconstruction, peaks in dRL fiber orientation density function (fODF) were estimated for each voxel. By this method, the proportion of fibers in each voxel at that point in each direction is estimated, providing information about complex fiber configurations. The dRL algorithm interpolated local fODF estimates and, from these data, tracking was carried out from seed points located at the vertices of a 2 mm × 2 mm × 2 mm grid. Streamlines were propagated from each seed point and tracking continued along the peak fODF with steps of 0.5 mm. Tracking continued until peak values fell below a threshold of 0.05 or the angle of the tract exceeded an angle of 45°. Streamlines with lengths over 500 mm or below 10 mm were excluded.

### Fornix

Fiber tracts were manually reconstructed using ExploreDTI version 4.8.3 (Leemans et al., 2009). A 3D reconstruction of the whole fornix was completed using the waypoint region of interest (ROI) protocol outlined by Christiansen et al. (2016). This involved the placement of ‘AND’, ‘OR’ and ‘NOT’ gates, according to Boolean logic, to isolate fibers of interest from whole brain tractography data. Guided by a series of anatomical landmarks, ROIs were manually drawn on native space color-coded FA maps (Pajevic and Pierpaoli, 1999) by operators blind to participants’ identity.

**Figure 1.**
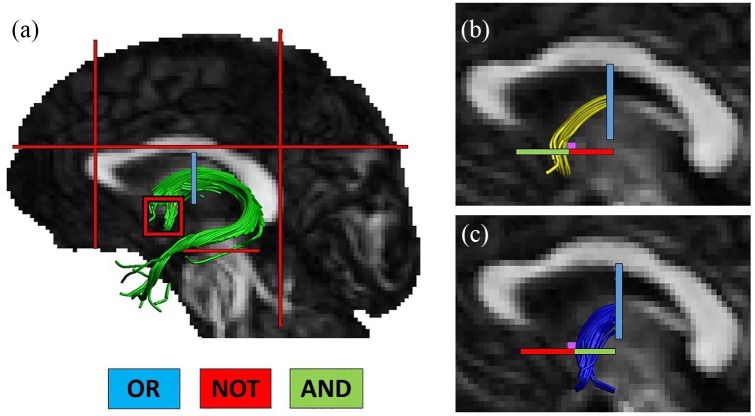
Example region of interest placements for tract extraction. (a) A depicts the whole fornix reconstruction, (b) depicts the precommissural fornix, and (c) depicts the postcommissural fornix. The location of the anterior commissure is indicated by the pink square in (b) and (c).

The fornix was reconstructed by placing an OR gate encompassing the entire fornix on a coronal slice six slices posterior to the slice containing the anterior commissure (see Figure 1). Captured fibers that were not consistent with the known path of the fornix were excluded from the reconstruction by the placement of a series of NOT gates located: on a coronal slice immediately anterior to the genu of the corpus callosum, on the axial slice immediately superior to the body of the corpus callosum, on a coronal slice immediately posterior to the splenium of the corpus callosum, on an axial slice at the upper limit of the pons, and on sagittal slices on either side of the fornix capturing the fibers of the anterior commissure.

### Precommissural and postcommissural fornix

To distinguish the precommissural fornix, the gates used to capture the whole fornix were utilised but with the addition of an AND gate placed on an axial slice anterior to the anterior commissure and a NOT gate placed on the same slice but posterior to the anterior commissure (see Figure 1). The same gates were utilised for the postcommissural fornix but with the placement of the additional AND and NOT gates reversed to capture those fibers running posterior to the anterior commissure. Only fibers up to the crus of the fornix were included in precommissural and postcommissural reconstructions in order to minimise overlap between the two fiber populations and to avoid fiber ‘jumping’. For this purpose, the ‘splitter’ tool within ExploreDTI 4.8.3 was utilised to extract only those fibers in each subdivision located anterior to the OR gate. Aside from minor modifications, this method followed that previously used to distinguish these fornix subdivisions (Christiansen et al., 2016). It was anticipated that the postcommissural subdivision would principally reflect the hippocampal–hypothalamic fibers in this pathway (Christiansen et al., 2016).

For each participant, the reconstructed fiber paths of the fornix and its precommissural and postcommissural subdivisions were used as masks and intersected with the whole brain maps of the eight microstructural metrics (FA, MD, ICSF, ISOSF, ODI, MPF, R_1_, *k_f_*) to extract mean tract scores.

### Statistical analysis

Statistical analyses were carried out in SPSS version 23 (IBM Corp, 2011). Before analyses were completed, cognitive scores and all microstructural metrics for each tract were inspected for outliers, defined as values lying beyond three standard deviations from the mean score. Outliers were removed from subsequent analyses.

The values for each of the metrics for the precommissural and the postcommissural fornix were compared. As the metrics were normally distributed, Pearson’s correlations were run in conjunction with paired-samples *t*-tests to determine both the extent to which values were correlated as well as the likelihood that values differed between the two tract subdivisions. Analyses then examined the relationship between the metrics for each tract and participants’ age to determine whether differential age effects were present across the two subdivisions. For all sets of tests, multiple comparisons were corrected with a false discovery rate (FDR) of 5% using the Benjamini–Hochberg procedure (Benjamini and Hochberg, 1995) and analyses showing potential differences between tracts were followed up with post hoc comparisons of correlation coefficients using directional Steiger Z-tests (Steiger, 1980; see also Lee and Preacher, 2013).

As three experimenters were involved in completing the manual tractography, intraclass correlation coefficients (ICCs) and their 95% confidence intervals were calculated to assess the reliability of metrics extracted for the tracts of interest. A two-way-mixed-effects model was used to calculate absolute agreement across raters. Single measure values are reported comparing metrics extracted from tractography performed by one experimenter (B.M.C.) with those extracted from tracts reconstructed by one of the other two experimenters (E.C., R.L.). There are currently no standardised interpretations for ICC values. Following the recommendations by Koo and Li (2016), we interpreted ICC values below 0.5 as poor, 0.5–0.75 as moderate, 0.75–0.9 as good, and above 0.9 as excellent.

For the cognitive data, exploratory factor analyses (EFAs) with unweighted least squares and orthogonal varimax rotation with a maximum of 5000 iterations for conversion were applied to the scores from all cognitive tests. This procedure sought to extract concise, meaningful dimensions from the large array of cognitive data with which to compare the precommissural and postcommissural pathways. Only variables with communalities of >0.4 were included in the final EFA (Osborne et al., 2008). After inspection of Cattell’s scree plot (Cattell, 1966), factors with an eigenvalue exceeding 2 were extracted and factor loadings exceeding 0.5 were considered for the interpretation of the factors.

The resulting cognitive factors were then entered as dependent variables into linear hierarchical regression models, which first tested for the effects of age, sex, and years of education and second for the effects of all microstructural indices in the whole fornix, the precommissural fornix, and postcommissural fornix in a stepwise fashion.

Of the 166 participants who completed cognitive assessment and MRI, data from 17 individuals were excluded. For 11 of these individuals, it was not possible to reliably reconstruct the fornix. Further to this, six individuals were excluded as the fornix was reconstructed but it was not possible to isolate one or both tract subdivisions. Subsequent comparisons showed that the 17 excluded participants were, on average, older than those retained (*t*(164) = –3.948, p = < 0.001), but did not differ with regard to years of education (p = 0.3), sex (p = 0.4), MMSE (p = 0.8), or NART-IQ (p = 0.55). For the remaining sample of 149 individuals, there were no significant differences in age (p = 0.85), years of education (p = 0.95), or NART (p = 0.95) between males and females. However, men performed slightly poorer on the MMSE [*t*(147) = 2.5, p = 0.014].

## Results

### Relationship between age and microstructural metrics of the whole fornix

For the fornix as a whole, seven outlying (3 + SD from mean) microstructural scores were identified. Two of these were obtained from a single participant. These outlying data points were excluded from further analysis, although non-outlying values from the same participants were retained. Significant correlations were observed between age and all metrics of interest, apart from ICSF (see Table 1).

**Table 1.** Pearson’s correlation coefficients (*r*) for the relationship between age and microstructural metrics of interest from reconstructions of the whole fornix.

Metric	*r*	*p*_B-Hadj_
FA	*–0.46*	<0.000001
MD	*0.30*	0.00023
ICSF	*–0.02*	0.817
ISOSF	*0.36*	0.00001
ODI	*0.23*	0.006
MPF	*–0.46*	<0.000001
R_1_	*–0.41*	<0.000001
*k_f_*	*–0.40*	<0.000001

FA: fractional anisotropy; MD: mean diffusivity; ICSF: intracellular signal fraction; ISOSF: isotropic signal fraction; ODI: orientation dispersion index; MPF: macromolecular proton fraction; *p*_B-Hadj_: Benjamini–Hochberg adjusted *p*-values; FDR: false discovery rate.

5% FDR corrected correlations are highlighted in italics.

### Fornix subdivisions

For the fornix subdivisions, nine individuals had at least one metric defined as an outlier. Six of these cases had more than one outlier. All outlying data points were excluded from further analysis, although non-outlying values from the same participant were retained.

**Figure 2.**
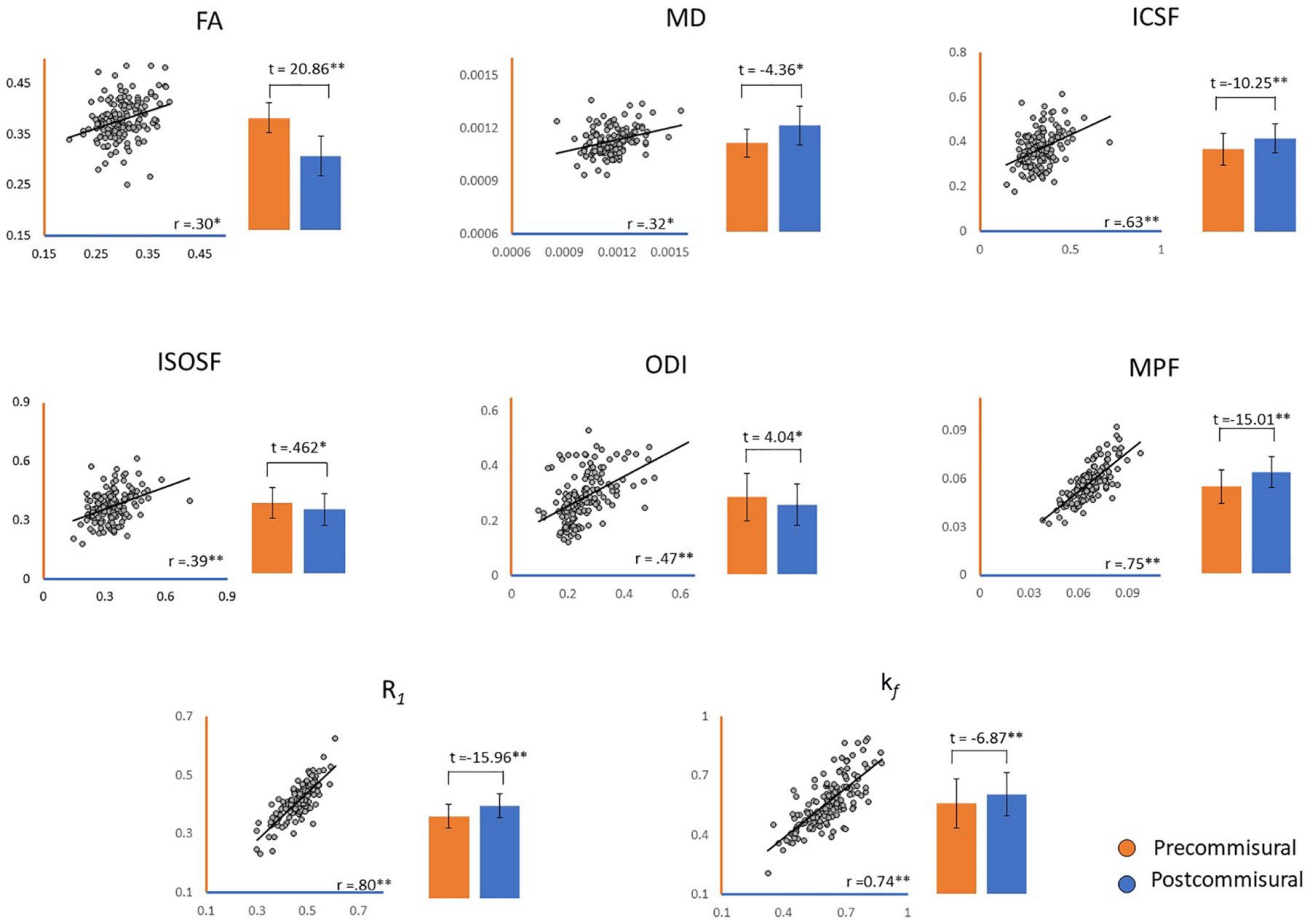
Correlations (*r*) between microstructural indices in the precommissural and the postcommissural fornix and between-tract comparisons for each microstructural index. FA: fractional anisotropy; MD: mean diffusivity; ICSF: intracellular signal fraction; ISOSF: isotropic signal fraction; ODI: orientation dispersion index; MPF: macromolecular proton fraction. *p_B-Hadj_ < 0.001; **p_B-Hadj_ < 0.000001.

Initial comparisons showed that for all measures, the metric values for the precommissural and postcommissural fornix were significantly correlated with each other (Figure 2). At the same time, all the actual metric values for the precommissural and postcommissural fornix differed significantly from each other (Figure 2).

### ICCs for microstructural metrics of precommissural and postcommissural fornices

Overall, the reliability of the metrics across the raters was good. For all tracts, greater agreement was seen for the postcommissural fornix than for the precommissural fornix (see Table 2) reflecting greater tractography consistency across raters for the postcommissural than the precommissural fornix. The ICCs across both tracts were particularly good for MPF, *k_f_*, R_1_, ICSF, and ODI, some of which could be considered excellent in the postcommissural fornix. The ICC for ISOSF in the postcommissural fornix was also good but in the precommissural fornix only moderate. However, poor agreement was observed for FA in both tracts and for MD in the precommissural fornix. Thus, while most metrics may be considered reliable, caution should be applied to the interpretation of FA and MD.

**Table 2.** Interclass correlation coefficients and their 95% confidence intervals for precommissural and postcommissural fornix.

	Precommissural fornix (95% CI)	Postcommissural fornix (95% CI)
FA	0.20 (–0.25–0.58)	0.12 (–0.31–0.51)
MD	0.42 (–0.02–0.73)	0.71 (0.40–0.88)
ICSF	0.75 (0.47–0.89)	0.83 (0.63–0.93)
ISOSF	0.58 (0.19–0.81)	0.76 (0.49–0.90)
ODI	0.78 (0.54–0.91)	0.90 (0.76–0.96)
MPF	0.73 (0.43–0.88)	0.96 (0.90–0.98)
R_1_	0.85 (0.67–0.94)	0.97 (0.91–0.99)
*k_f_*	0.76 (0.49–0.90)	0.86 (0.68–0.94)

CI: confidence interval; FA: fractional anisotropy; MD: mean diffusivity; ICSF: intracellular signal fraction; ISOSF: isotropic signal fraction; ODI: orientation dispersion index; MPF: macromolecular proton fraction.

### Relationship between age and microstructural metrics of precommissural and postcommissural fornices

**Figure 3.**
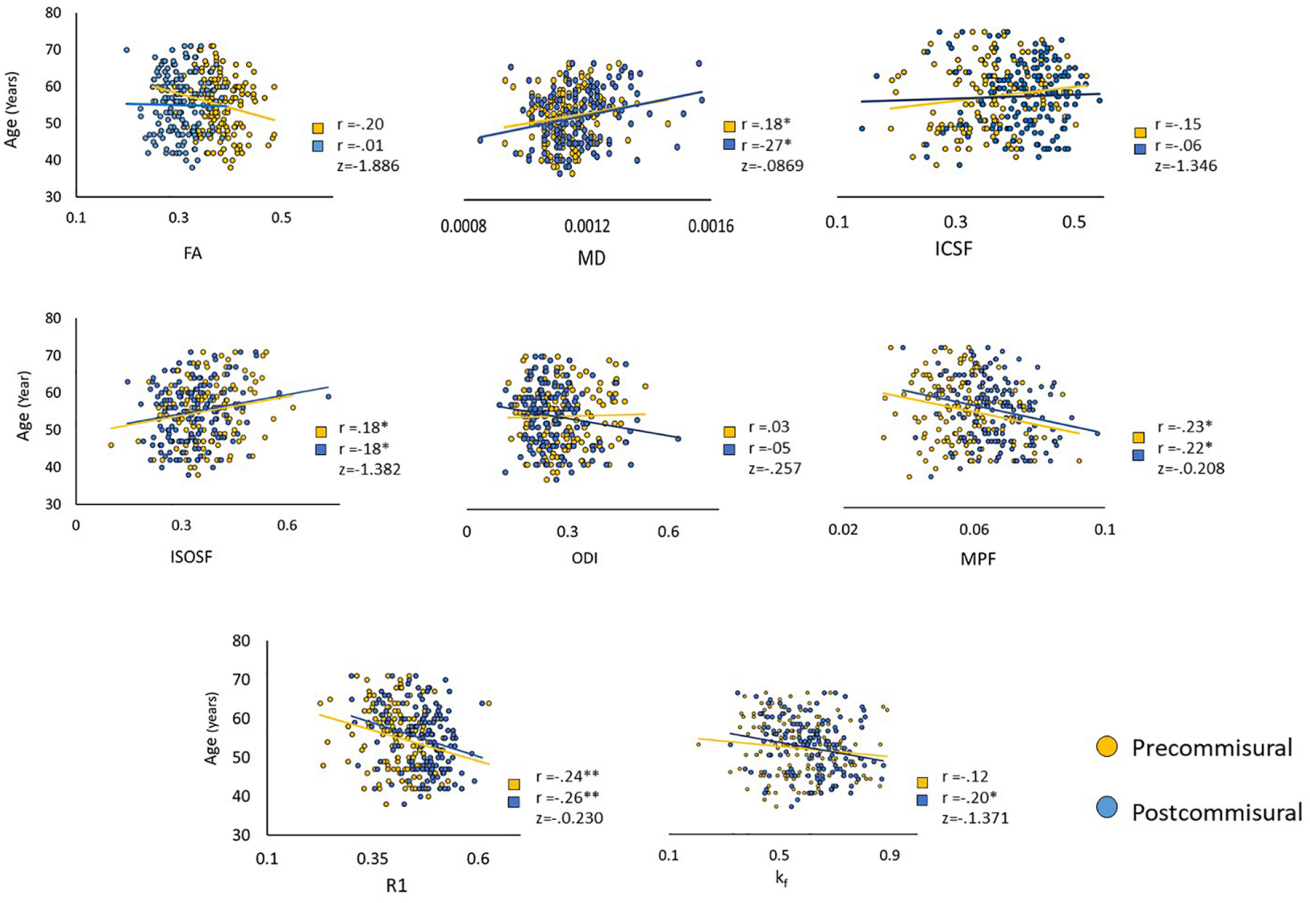
Correlations (*r*) between metrics of interest and age, as well as a comparison of correlation coefficients across precommissural and postcommissural fornix (z). FA: fractional anisotropy; MD: mean diffusivity; ICSF: intracellular signal fraction; ISOSF: isotropic signal fraction; ODI: orientation dispersion index; MPF: macromolecular proton fraction.

There were significant positive correlations between age and MD or ISOSF and significant negative correlations between age and MPF or R_1_ in both precommissural and postcommissural fornices, and these correlations did not differ between the pathways (Figure 3). An age-related reduction was also observed for *k_f_* in the postcommissural fornix.

No significant associations were observed between age and FA or ODI for either the precommissural or the postcommissural fornix (Figure 2), despite both indices being associated with age when the whole fornix was considered (Table 1). Finally, as with the whole fornix, neither pathway showed an age-related correlation with ICSF.

### Relationship between white matter metrics and cognitive performance

After exclusion of variables with communalities of <0.4 (Stroop, Hampshire Tree, Recall of List B in RAVLT, Four Mountains Test, copying of Rey figure, intra-dimensional shift, and reversal for one dimension), and only including factors with an eigenvalue of >2, EFA led to seven factors, explaining together 49% of the variance in the cognitive data (Table 3). The first factor captured ‘Verbal Recall’ with high loadings on the RAVLT. The second factor captured elements of ‘Motor speed and planning’ with high loadings on first move latencies in the hidden platform and motor control blocks as well as total latencies in the motor control condition of the vMWMT. A third factor captured ‘Spatial Navigation’ with high loadings on path length and total latencies in the hidden platform conditions of the vMWMT. The fourth factor captured ‘Attention Set’ with high loadings on the intra-dimensional components of the IDED task. The fifth factor captured ‘Visuospatial Memory’ with loadings on spatial span and immediate and delayed recall of the Rey Figure. The sixth ‘Working Memory’ factor loaded highly on digit span and spatial search, and the seventh ‘Paired Associate Learning’ factor on the object-location PAL task.

**Table 3.** Rotated factor matrix of the exploratory factor analysis within the cognitive data (rotation methods: Varimax with Kaiser normalisation).

Cognitive scores	Verbal recall	Motor speed and planning	Spatial navigation	Attention set	Visuospatial memory	Working memory	Paired associate learning
Rey Auditory Verbal Learning Test							
List A first IR	0.73						
List A second IR	0.87						
List A third IR	0.83						
List A fourth IR	0.81						
List A fifth IR	0.73						
List A sixth IR	0.83						
List A DR	0.84						
Virtual Morris Water Maze Task							
FM Block2		0.70					
FM Block3		0.66					
FM Block4		0.63					
FM Block5		0.61					
FM Block6		0.69					
TL Block2			0.56				
TL Block5			0.54				
TL Block6		0.72					
PL Block2			0.64				
PL Block3			0.51				
PL Block4			0.56				
PL Block5			0.67				
Rey complex figure							
IR					0.56		
DR					0.63		
Spatial span							
Maximum					0.66		
Mean					0.66		
Intra-dimensional and extra-dimensional attention shift							
ID two dimensions				0.57			
ID overlapping features				0.63			
ID reversal				0.61			
ID shift				0.52			
Total duration				0.82			
Digit span							
Maximum						0.74	
Mean						0.73	
Spatial search							
Maximum						0.53	
Mean						0.50	
Paired associate learning							
Maximum							0.90
Mean							0.95

DR: delayed recall; ED: extra-dimensional; FM: first move latencies; ID: intra-dimensional; IR: immediate recall; PL: path length; RT: reaction time; TL: total latency.

Loadings of >0.5 are displayed.

### Hierarchical regression analyses

To assess which microstructural indices accounted for differences in the cognitive components, hierarchical regression analyses were carried out that first accounted for the effects of age, sex, and education before testing for the effects of all microstructural indices in a stepwise fashion. For the Verbal Recall factor, 13% of the data (F(3, 98) = 4.9, p = 0.003) were explained by the first model with a significant contribution from sex (beta = 0.36, p_B-Hadj_ < 0.001). For the Motor Speed and Planning factor, 18% of the data (F(3, 98) = 7.1, p < 0.001) were accounted for by the first model alone with age as significant predictor (beta = 0.41, p_B-Hadj_ < 0.001). Adding the microstructural indices did not improve the fit of the regression models for either factors.

For the Visuospatial Memory factor, the first model explained 8% of the data (F(3, 98) = 2.8, p = 0.045) with a contribution from age (beta = –0.26, p = 0.01). Adding the microstructural indices increased the model fit significantly (ΔR^2^ = 0.04, F(1, 97) = 4.9, p = 0.03). The final model accounted for 12% of the data (F(4, 97) = 3.4, p = 0.012) with contributions from precommissural fornix ISOSF (beta = –0.22, p = 0.03) and age (beta = –0.24, p = 0.019). However, these variables did not survive 5% FDR correction.

For the Paired Associate Learning factor, the first model was non-significant (p = 0.55). Adding the microstructural components, increased the fit of the model (ΔR^2^ = 0.04, F(1, 96) = 4.4, p = 0.038). The final model explained 11% of the data (F(5, 96) = 2.4, p = 0.042) with a significant contribution from postcommissural ODI (beta = –0.29, p_B-Hadj_ = 0.02) and a trend for postcommissural ISOSF (beta = 0.23, p = 0.038, p_B-Hadj_ = 0.09).

There were no significant results for the regression models for the Spatial Navigation (p = 0.06), Attention Set (first model p = 0.37, second model p = 0.13), and Working Memory factors (first model p = 0.28, second model p = 0.07).

## Discussion

The fornix and its major subdivisions were studied in a healthy aging cohort and it proved possible to distinguish the precommissural from the postcommissural fornix (see also Chen et al., 2015; Christiansen et al., 2016; Yeo et al., 2013). Only for a small minority of participants (~10%), the subdivisions could not be reconstructed, a problem most apparent in older participants. A central goal was to go beyond conventional DTI indices as they are difficult to interpret in terms of biological tissue properties (Beaulieu and Allen, 1994; De Santis et al., 2014). Instead, we studied the white matter microstructure of the fornical subdivisions with multi-parametric indices from multi-component diffusion, and more myelin-sensitive indices from qMT and T_1_-relaxometry. These indices were found to have better reproducibility than conventional FA and MD (Table 2; see also Koller et al., 2019).

### Whole fornix

Consistent with, but also extending previous studies, the fornix displayed age-associated changes for almost all of the metrics, with FA showing particularly strong effects (see also Chen et al., 2015; Gazes et al., 2019; Ly et al., 2016; Sala et al., 2012). The sole exception was the ICSF, an estimate of intracellular restricted diffusion that may give a proxy measure of axon density assumed to vary with the number and the size of axons (Assaf et al., 2004; Zhang et al., 2012). In a related CARDS cohort study (Metzler-Baddeley et al., 2019) that used automatic, rather than manual, reconstructions of the fornix, aging was again associated with fornix differences in all NODDI and qMT metrics except for ICSF. These close similarities, alongside the results from other studies (Chen et al., 2015; Gazes et al., 2019; Sala et al., 2012), highlight the robustness of aging upon the large majority of measures of whole fornix microstructure.

Some of the strongest age-related effects came from measures known to be sensitive to myelin in white matter (MPF, R_1_, *k_f_*). This pattern accords with a post-mortem study of rhesus monkeys, which found an age-related loss of myelinated axons and an increase in altered myelin sheaths, but preservation of unmyelinated fibers (Peters et al., 2010). The present findings also build on other imaging analyses, which indicate that fornix maturation peaks in late adolescence (Douet and Chang, 2015) with subsequent volume reductions in healthy aging (e.g. Amaral et al., 2018; Douet and Chang, 2015) that partly reflect a loss of myelinated fibers. The overall conclusion from present and previous research is that myelinated fornix fibers are particularly sensitive to aging. There was, however, no apparent age-related effect on fornix ICSF (see also Metzler-Baddeley et al., 2019), suggestive of a preservation of apparent axon density.

#### Precommissural versus postcommissural fornix

While the precommissural reconstructions should contain septal fibers alongside hippocampal projections to the ventral striatum, basal forebrain, medial and orbital frontal cortices, the postcommissural reconstructions should principally contain projections to the hypothalamus, including the mammillary bodies (Christiansen et al., 2016). Despite the many hippocampal–thalamic fibers in the postcommissural fornix, it was presumed that they would comprise far less of the reconstructions given their diffuse, short trajectories on reaching the diencephalon (Mathiasen et al., 2019). It is also presumed that the fibers in the two fornix subdivisions originate from overlapping parts of the hippocampal formation (Aggleton, 2012), only fully separating as the body of the fornix approaches the columns.

All eight diffusion indices derived in this study were highly inter-correlated between the two tract subdivisions (Figure 2). This is consistent with previous observations of intra-individual correlations between microstructural metrics across white matter tracts (Penke et al., 2010). However, despite the many correlations, the absolute values of all diffusion metrics differed between the two fornix subdivisions (Figure 2). The NODDI and qMT metrics in this study suggest that the precommissural fibers may perhaps be smaller (ICSF) and have lower myelin-related properties (MPF, *k_f_*, R_1_) and, hence, a higher free water component (ISOSF) than postcommissural fibers. Precommissural fibers also showed higher axon orientation dispersion (ODI). This pattern of differences may partly reflect how axons in the precommissural fornix disperse in order to reach their multiple targets while those in the descending postcommissural fornix remain largely compact until reaching the posterior hypothalamus (Mathiasen et al., 2019; Poletti and Creswell, 1977). Nevertheless, FA was higher, and MD lower, in the precommissural relative to the postcommissural fornix, despite a previous report of higher FA in the postcommissural than precommissural fornix (Yeo et al., 2013). This apparent inconsistency may arise from the use of different seed ROIs, most notably for the postcommissural fornix, allied to its greater curvature and how that part of the fornix passes through many diffuse pathways in the rostral hypothalamus. However, given the poor reproducibility of FA and MD in both tracts, these results should be interpreted with caution and may not accurately reflect the microstructural differences between precommissural and postcommissural fornices.

Similarly, despite age being strongly correlated with whole fornix FA, this same correlation was not significant for either the precommissural or postcommissural fornix. This was likely due to the poor reproducibility of FA in both tracts. Another interpretation is that age-related changes in fornix FA are more strongly driven by alterations to the body of the fornix. Consistent with this suggestion, age correlations in FA, RD, and AD were found to be limited to the caudal body and left crus of the fornix (Chen et al., 2015). Likewise, Christiansen et al. (2016) failed to find a significant age relationship between FA and the anterior body of the fornix, which included the columns. Meanwhile, in a study including even older participants (Jang et al., 2011), decreasing FA with age was found in the body, crus, and columns of the fornix, suggesting that all parts of the tract can contribute. Taken together, the implication is that whole fornix provides the most consistent target for associating age with FA, when using sufficient age ranges.

Christiansen et al. (2016) previously reported age correlations with RD, MD, and AD in the postcommissural but only trends in the precommissural fornix. Here, with appreciably larger sample size, we found evidence for age-related increases in MD and ISOSF and reductions in MPF and R_1_ for both tracts. Age-related reductions in *k_f_* were only observed for the postcommissural fornix, and no age correlations were present for ICSF or ODI. Consistent with the findings for the whole fornix, this pattern suggests that age-related reductions in apparent axon myelin rather than in apparent axon density or orientation may underpin age-related tissue loss in both subcomponents of the fornix.

The final goal was to consider cognitive functions. Based on connectivity, the postcommissural fornix might be expected to be the more strongly associated with changes in long-term memory as it contains projections from the hippocampus to interlinked structures (the mammillary bodies and anterior thalamic nuclei) repeatedly implicated in human diencephalic amnesia and spatial memory in rodents (Aggleton and Brown, 1999; Carlesimo et al., 2011; Harding et al., 2000; Vann and Nelson, 2015). Additional evidence comes from correlations between mammillary body volume and performance on tests of recall (Tsivilis et al., 2008; Vann et al., 2009). Meanwhile, the precommissural fornix provides the direct route from the hippocampus to the prefrontal cortex (Aggleton et al., 2015), suggesting an involvement in executive or control processes (Miller and Cohen, 2001). The basal forebrain–hippocampal connections carried by the precommissural fornix contribute to hippocampal theta and acetylcholine and may therefore be linked to attention and spatial flexibility (Baxter and Chiba, 1999).

Consistent with its hippocampal–diencephalic connections, the postcommissural fornix was associated with the Paired Association Learning factor. A parallel can be seen with evidence that object-in-place learning by monkeys is dependent on the integrity of the fornix and mammillary bodies (Gaffan, 1994; Parker and Gaffan, 1997), that is, the postcommissural fornix. However, as spatial learning relies on a network of medial temporal lobe brain regions including the hippocampus (Burgess et al., 2002), it is not surprising that postcommissural fornix contributions did not completely explain all of the variation in the PAL data, nor was it associated with other spatial cognitive factors.

Variations in Verbal Memory performance was best predicted by sex (with women performing better in the RAVLT than men, see Metzler-Baddeley et al., 2019a) and variation in motor speed and planning by age. There was a trend for precommissural microstructure to predict variation in Visuospatial Memory, with high loadings on spatial span and the free recall of the Rey figure. While this result was not significant after FDR correction, precommissural fornix contributions to strategic aspects of visuospatial recall would be consistent with its connectivity to prefrontal cortex regions (Eichenbaum, 2017).

In summary, these findings suggest that the postcommissural fornix is involved in aspects of visual spatial learning, a result that accords with the diencephalic regions innervated by this fornix subdivision. Nevertheless, there is evidence that in the absence of this postcommissural pathway, other networks can compensate (Vann et al., 2011). Indeed, the lack of clear fornix subdivision dissociations could indicate that both tracts may contribute, albeit in different ways, to common cognitive tasks.
